# Fabrication and evaluation of a sulfacetamide-loaded niosomal gel for the treatment of ocular bacterial infections

**DOI:** 10.1371/journal.pone.0362096

**Published:** 2026-11-05

**Authors:** Neda Sistani Karampour, Pouya Dolati, Anayatollah Salim, Maryam Abedini Baghbadorani, Maryam Keykhaee, Somayeh Handali, Eskandar Moghimipour

**Affiliations:** 1 Marine Pharmaceutical Science Research Center, Ahvaz Jundishapur University of Medical Sciences, Ahvaz, Iran; 2 Nanotechnology Research Center, Ahvaz Jundishapur University of Medical Sciences, Ahvaz, Iran; 3 Department of Toxicology, School of Pharmacy, Ahvaz Jundishapur University of Medical Sciences, Ahvaz, Iran; 4 Medical Biomaterials Research Centre (MBRC), Tehran University of Medical Sciences, Tehran, Iran; Dubai Pharmacy College, UNITED ARAB EMIRATES

## Abstract

Topical ocular drug delivery is often restricted by rapid precorneal clearance, which can decrease drug residence time and bioavailability at the ocular surface. The purpose of the study was to develop a sulfacetamide-loaded niosomal gel (SA-Nio-Gel) to enhance ocular drug delivery. Thin film hydration method was used for the preparation of SA niosomes, employing nonionic surfactants (Span 60 and Span 80) and cholesterol. The optimized niosomal formulation was then incorporated into a Carbopol 934 gel base to prepare the final SA-Nio-Gel formulation. The optimized niosomes exhibited encapsulation efficiency of 76% ± 1.5 and a mean particle size of 129.7 ± 2.52 nm. Rheological analysis of the SA-Nio-Gel demonstrated pseudoplastic behavior, characterized by a reduction in viscosity with increasing shear rate. Biocompatibility evaluation indicated that the formulation was non-toxic. *In vitro* release profiles showed a slow and sustained SA release from the developed formulation. Furthermore, microbiological assays confirmed the potent antibacterial activity of the SA-Nio-Gel against *S. aureus.* Compared to the SA solution and SA-Nio, the SA-Nio-Gel formulation significantly minimized precorneal drainage and improved drug penetration. These results indicate that the niosomal gel-based system could be a promising platform for the ocular delivery of SA, which provides sustained release and enhanced drug permeability.

## Introduction

Bacterial eye infections, particularly conjunctivitis, can spread to deeper layers of the eye and lead to serious complications if not effectively treated. Tight epithelial barriers such as cornea, the constant flow of tears and the blinking reflex prevent the persistence of microorganisms in the eye. Nevertheless, factors such as corneal injury, allergic reactions, overuse of contact lenses, and systemic diseases such as diabetes can make the eye susceptible to infections [1–4].

*Conventional* eye drops are widely used for treating various ophthalmic problems; however, they have several drawbacks. The eye’s natural defense mechanisms, such as blinking and tear flow, rapidly clear the drops from the eye [5,6]. Only about 5% of the administered drug penetrates the intraocular tissues, while the majority is lost due to drainage. This low ocular bioavailability necessitates frequent dosing, which can reduce patient compliance. Importantly, early and effective treatment of bacterial infections is crucial to prevent the infection from spreading to deeper parts of the eye [7,8].

Sulfacetamide sodium (SA) is a synthetic sulfonamide with strong antibacterial activity that inhibits bacterial growth by competing with para-aminobenzoic acid to prevent the production of folic acid. SA eye drop is often employed to treat conjunctivitis and other eye infections caused by bacteria like *Staphylococcus aureus* (*S. aureus*), *Escherichia coli* (*E. coli*), *Streptococcus pneumoniae* (*S. pneumoniae*) and *Klebsiella* species [1,7,9]. A major limitation of commercial SA eye drops is the need for frequent dosing, typically every 2–3 h, which leads to patient non-compliance [9], highlighting the need for ocular drug delivery systems capable of prolonging drug residence time and maintaining drug availability at the ocular surface.

Several approaches have been widely investigated to improve drug bioavailability in the eye. One of these strategies is the development of drug delivery systems such as liposomes [10,11], micelles [12,13], microemulsions [14] and niosomes [15,16].

Niosomes are nanosized and spherical vesicles composed of nonionic surfactants that form a lipid bilayer, enabling the encapsulation of both hydrophobic and hydrophilic drugs. In recent years, they have received considerable attention for ocular drug delivery due to their ability to prolong drug release, enhance the retention of the drug in the eye, protect the loaded drug from the enzymes present on the corneal surface and improve drug solubility and stability. Moreover, niosomes are non-toxic, biocompatible, simple and cost-effective to fabricate, and do not require special storage conditions, making them an ideal nanocarrier for ocular drug delivery [2,17,18]. Despite these advantages, nanovesicles are still rapidly washed away and eliminated by tears and blinking. To address this issue, mucoadhesive polymers can be incorporated into the formulation to bond with the mucin layer and extend the contact time with the cornea [19]. Carbopol, a mucoadhesive polymer, has been gained more attention in ocular drug delivery because of its high viscosity at low concentration and minimal toxicity [20]. This polymer contains a large number of carboxyl groups that can form hydrogen bonds with carboxylate groups on the carbohydrate side chains of mucin, forming a strong network with the mucous membrane and enhancing mucoadhesive properties [21].

As illustrated in Fig 1, the purpose of the study was to develop a niosomal gel containing SA (SA-Nio-Gel) to improve ocular drug delivery. This formulation is designed to combine the sustained release and drug protection properties of niosomes with the mucoadhesive properties of Carbopol gel, in order to improve drug retention time, reduce dosing frequency, and ultimately increase patient satisfaction and treatment efficacy.

**Fig 1:**
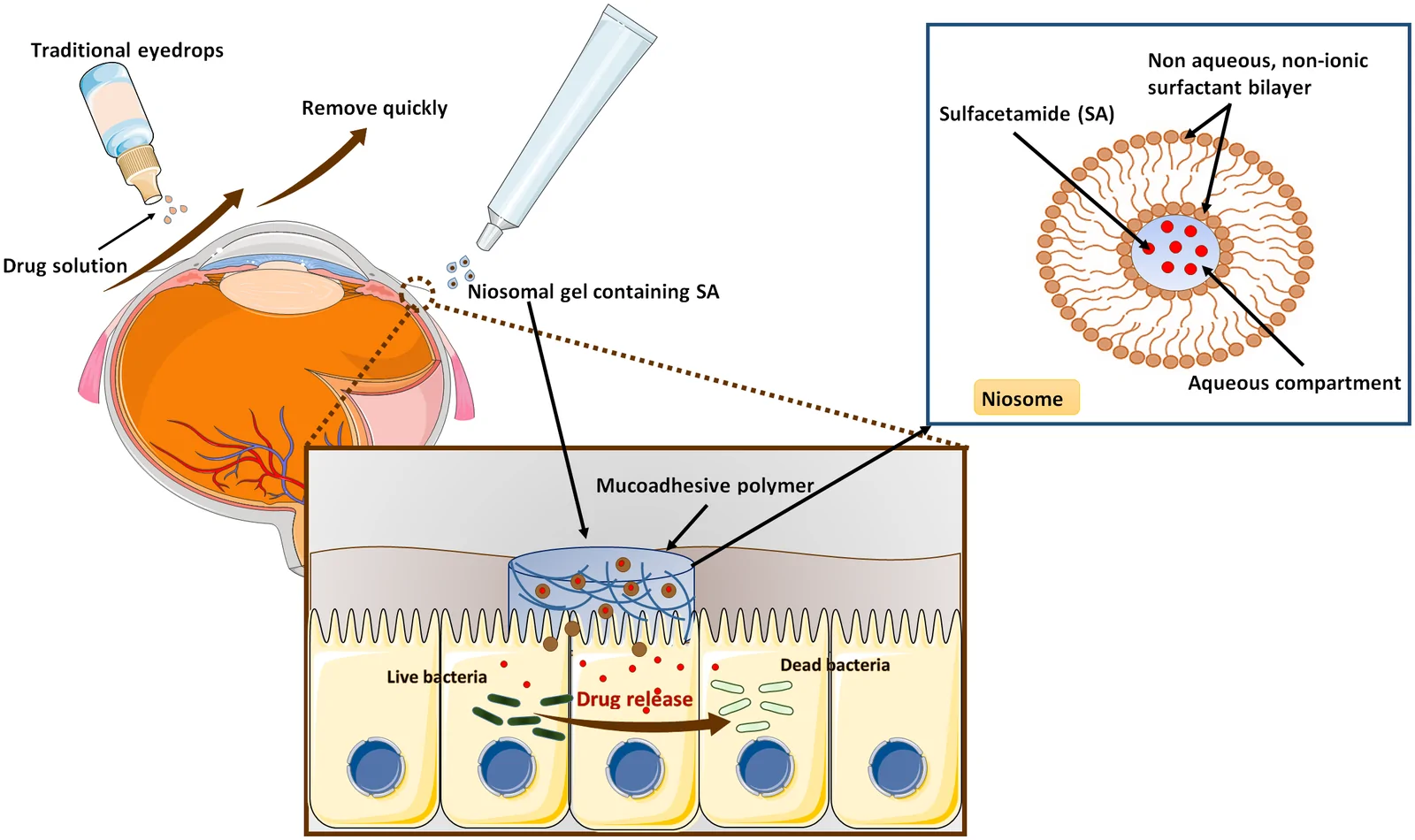
A schematic illustration of niosomal gel formulation for enhanced ocular delivery of Sulfacetamide (SA).

## Materials and methods

Sulfacetamide was obtained from Sinadarou Co. (Iran). Span 60 (CAT NO: 840121), Span 80 (CAT NO: 840123**)**, cholesterol (CAT NO: 228111), Carbopol 934, agar medium and benzalkonium chloride (CAT NO: 137124) were purchased from Merck (Germany). Dulbecco’s modified eagle’s medium (DMEM) and fetal bovine serum (FBS) were acquired from Gibco (the USA). Hu02 cells, *S. aureus* and male rabbits were provided from the Pasteur Institute of Iran. Dialysis membrane, penicillin-streptomycin and 3-(4,5-dimethyl-2-thiazolyl)-2,5-diphenyl tetrazolium bromide (MTT) were purchased from Sigma Aldrich (Germany). Other chemicals and solvents were of analytical grade and obtained from Merck (Germany).

### Preparation of SA loaded niosomes

SA-loaded niosomes (SA-Nios) were prepared by a thin film hydration method. Briefly, surfactants (Sapn 60 and Span 80) and cholesterol (50 mg) were dissolved in chloroform and diethyl ether (1:1) under continuous stirring at 210 rpm. The low and high values of surfactants were selected according to the preliminary studies and literature review (Table 1). After removal of the solvent in a rotary evaporator (Heidolph, Germany), the thin lipid film was hydrated with 10 mL phosphate buffer saline (PBS, pH 7.4) containing SA (20%) by sonication in a water bath (Elma, Germany) for 20 min at 60 ^◦^C [22]. Then, SA-Nios were separated by centrifugation at 15000 rpm for 30 min (MPW-350R, Poland) and subsequently freeze dried at −50°C (2 Pa) for 24 h (Operon, Korea).

**Table 1:** Composition, EE % and particle size (nm) of different noisome formulations (n = 3).

Formulation No.	Surfactant (mg)		Cholesterol (mg)	Surfactant/ Cholesterol	EE %	Particle size (nm)
	Span 60	Span 80				
F1	–	50	50	1:1	48 ± 6	145.7 ± 1.53
F2	–	100	50	2:1	66 ± 2.5	134.3 ± 3.79
F3	50	–	50	1:1	60 ± 2	154.7 ± 2.08
F4	100	–	50	2:1	56 ± 4.5	163.3 ± 3.06
F5	**50**	**50**	**50**	**2:1**	**76 ± 1.5**	**129.7 ± 2.52**

### Determination of entrapment efficiency (EE%)

The EE% values of the obtained niosomes were evaluated by an indirect method. The niosomal dispersions were centrifuged at 15000 rpm and at 25 ^◦^C for 30 min. The amount of SA in the supernatant was determined using a UV spectrophotometer (Biowave-2, WPA, The UK) at 271 nm [1]. Then, the EE% was calculated according to the following Eq. 1:


$$\begin{array}{c} EE\%=({SA}_{t}-{SA}_{f}/{SA}_{t})\times 100 \end{array}$$ (1)

where, SA_t_ is the amount of drug initially added to the formulation and SA_f_ is the amount of the free drug in the supernatant after centrifugation [23].

### Determination of particle size and morphology of niosomes

The niosomes particle size was determined using a particle sizer (Qudix, scatterOscope I, Korea) at 25 ^°^C. Before measurement, samples were diluted with distilled water. For morphological evaluation, the niosomal suspension was dried and coated with a thin gold layer and then examined using a scanning electron microscope (SEM, VEGA3, TESCAN, Czech Republic).

### Preparation of niosomal gel containing SA

For preparation gel, Carbopol 934 (1% W/V) was dispersed in PBS under constant stirring at 300 rpm at 25 ^°^C. After complete dissolution, the optimized niosomal formulation was then incorporated into the gel, followed by the addition of benzalkonium chloride (0.005% w/) as a preservative.

### Rheological studies

The rheologic behavior of the samples was determined using a Brookfield viscometer (DV-I, The UK) with No. 64 spindle. The viscosity of the samples was measured at various shear rates for 1 min at room temperature. The obtained data were plotted as rheograms and the flow behavior was determined by fitting the curves to Newtonian and non-Newtonian models.

### Spreadability test

The spreadability of the formulations was determined by placing 1 g of gel between two glass slides. A standard weight about 100 g was applied on the upper slide for 1 min to assess the spread ability and diameters of the spread circles were then recorded.

### In vitro release

*In-vitro* drug release of SA solution (Sol), SA-Nio and SA-Nio-Gel was evaluated using Franz diffusion cells at 37 ± 1 ^°^C with stirring at 100 rpm. 1 mL of each formulation was placed on the dialysis membrane (MWCO 12 kDa). At specific time intervals (2, 4, 6, 8 and 10 h), 2 mL of release medium was withdrawn and an equal volume of fresh medium was added to maintain sink condition. The concentration of released drug was assayed using a UV spectrophotometer at 271 nm.

### *In vitro* cell viability assay

The cytotoxicity of optimized formulation was determined on Hu02 cells (a normal fibroblast cell line) by MTT assay. Cells were cultured at 37 ^°^C, 5% CO_2_ and 95% relative humidity in DMEM supplemented with 10% FBS and 1% penicillin-streptomycin. Cells were transferred to 96-well culture plate at 1 × 10^4^ cells per well 24 h before treatment. The optimized formulation was incubated in culture medium at concentration of 25 and 50 mg/mL for 24 h at 37 ^°^C. 100 µL of the extract was then transferred into each well and incubated for 24 h at 37 ^°^C. After treatment, the medium was removed and 50 μL of MTT solution (5 mg/mL in PBS) was added to each well, followed by incubation for 4 h at 37 ^°^C. Afterwards, 150 μL of dimethyl sulfoxide (DMSO) was added to dissolve the formazan and the plate was gently shaken for 20 min at room temperature [24]. Cell viability was determined by measuring absorbance at 570 nm using an ELISA plate reader (Synergy, BioTek Instruments Inc., Germany). The viability percentage was calculated using the following Eq. 2:


$$\begin{array}{c} Cell\text{\textasciitilde{}}viability\text{\textasciitilde{}}\%=({Abs}_{sample}/{Abs}_{\text{control}})\times 100 \end{array}$$ (2)

### *In vitro* antibacterial activity

The antimicrobial activity of SA-Nio and SA-Nio-Gel was evaluated using agar well diffusion method. A fresh bacterial culture of *S. aureus* was prepared and the bacterial suspension was adjusted to a turbidity equivalent to 0.5 McFarland standard. The suspension was then spread on the surface of the agar plates using a sterile swab. Wells with a diameter of 5 mm were created on the agar surface and 200 μL of each formulation was inoculated into the wells. The plates were then incubated at 37 ^°^C for 24 h. Following incubation, the **zones of inhibition** were measured.

### *Ex vivo* evaluation of ocular retention

All the animal experiments were conducted according to the guideline of the Animal Ethics Committee Jundishapur University of Medical Sciences, Ahvaz, Iran (ref no. IR.AJUMS.ABHC.REC.1401.089). A total of 15 adult male rabbits (weight, 2- 2.5 kg) were used in the study and randomly divided into three groups (n = 5 per group). All animals were housed under controlled environmental conditions (22 ± 2 ^°^C and humidity of 55 ± 5%) with free access to food and water. The animals were euthanized by the administration of sodium pentobarbital (100 mg/kg) before tissue collection. Following euthanasia, ocular mucosa was cut in 2 × 2 cm pieces and mounted on inclined glass slides. Then, a fixed amount of Sol, SA-Nio and SA-Nio-Gel were placed on each tissue piece*.* After exposure, the tissues were washed with simulated lacrimal fluid at a constant rate of 1.2 mL/min for 10 min. The washing solution was collected at 2 min intervals, and the drug concentration was determined using a UV spectrophotometer at 271 nm [7]*.*

### Statistical analysis

All results were presented as mean ± SD and one way ANOVA test was used for comparison of the data using SPSS software. The *p* value of less than 0.05 was considered statistically significant.

## Results and discussions

Drug delivery to ocular tissues is restricted by several physiological barriers, such as rapid tear turnover and the corneal barrier [25,26]. The results of the present study established that the SA-loaded niosomal gel system may efficiently overcame these limitations. Our data showed that the optimized formulation achieved sustained drug release over 10 h and enhanced drug penetration across ocular mucosa. These findings indicate that the prepared niosomal gel may reduce the frequency of administration and improve patient adherence to treatment.

### Preparation of SA loaded niosomes

Cholesterol plays an important role in the structural integrity and stability of niosomes. In this study, equal amounts of cholesterol were used for preparation of all formulations. It increases the stability of the bilayers through hydrogen bonds formed between the hydroxyl groups of cholesterol and the alkyl chains of the surfactant molecules. These interactions enhance membrane cohesion and limit the mobility of acyl chains within the bilayer [27]. According to the findings (Table 1), the optimal formulation is F5 (Span 60: Span 80 in a 1:1 ratio, with a total surfactant/cholesterol ratio of 2:1), which resulted in the highest encapsulation efficiency (76% ± 1.5) and the smallest particle size (129.7 ± 2.52 nm). Span 60 has a saturated alkyl chain and high phase transition temperature, which results in the formation of a rigid bilayer that reduces drug leakage and improves encapsulation efficiency [27–29]. In contrast, Span 80 has higher hydrophobicity and membrane flexibility due to its lower HLB value than other Span surfactants, causing greater membrane curvature and resulting in the formation of smaller vesicles [30,31]. Therefore, combining Span 60 and Span 80 in the F5 formulation can create a synergistic balance between the firmness and flexibility of the membrane. Span 60 provides structural integrity and increases drug retention, while Span 80 contributes to membrane fluidity and elasticity, which enhances the formation of smaller vesicles.

### Determination of particle size and morphology of niosomes

The SEM image and particle size distribution (Fig 2A and 2B) confirm the formation of monodisperse spherical nanocarriers. Smooth surfaces and spherical morphology are crucial for structural integrity and controlled drug release [32]. Moreover, for ophthalmic applications, nanoparticles should exhibit a narrow size distribution, as this parameter significantly contributes to improving ocular absorption [33].

**Fig 2:**
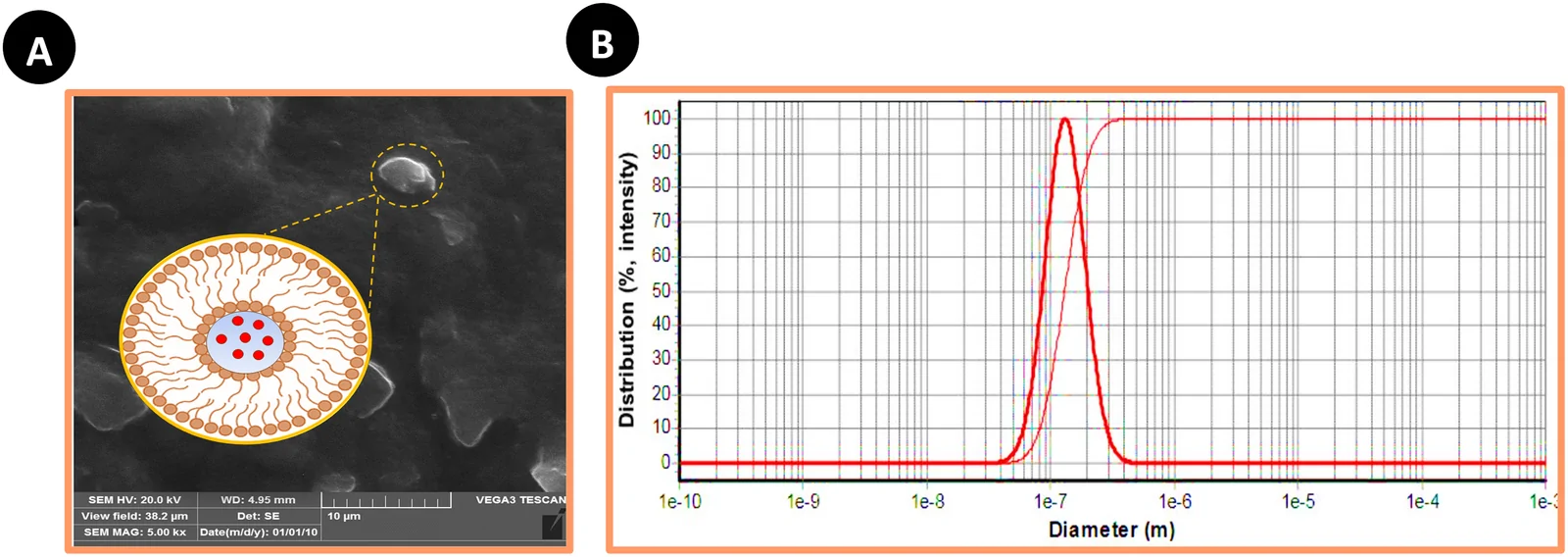
A) SEM image and B) particle size distribution of SA loaded niosomes.

### Rheological studies

As shown in Fig 3A, blank Gel and SA-Nio-Gel exhibited a pseudoplastic behavior as the viscosity decreased with an increase in shear rate [34]. For topical ophthalmic formulations, pseudoplastic behavior is considered a desirable property [35]. Because during blinking, the eye creates high shear stress, causing the gel to become less viscous due to its pseudoplastic behavior. This reduction in viscosity facilitates its uniform spreading over the corneal surface. When blinking stops and shear stress decreases, the gel regains its viscosity, allowing it to remain on the surface of the eye and prevent rapid removal by tear fluid [36,37]. These results are in-line with those obtained by Xingqi et al. and Cao et al., who showed that gels with pseudoplastic behavior improved drug dispersion and ocular absorption on the eye surface [38,39].

**Fig 3:**
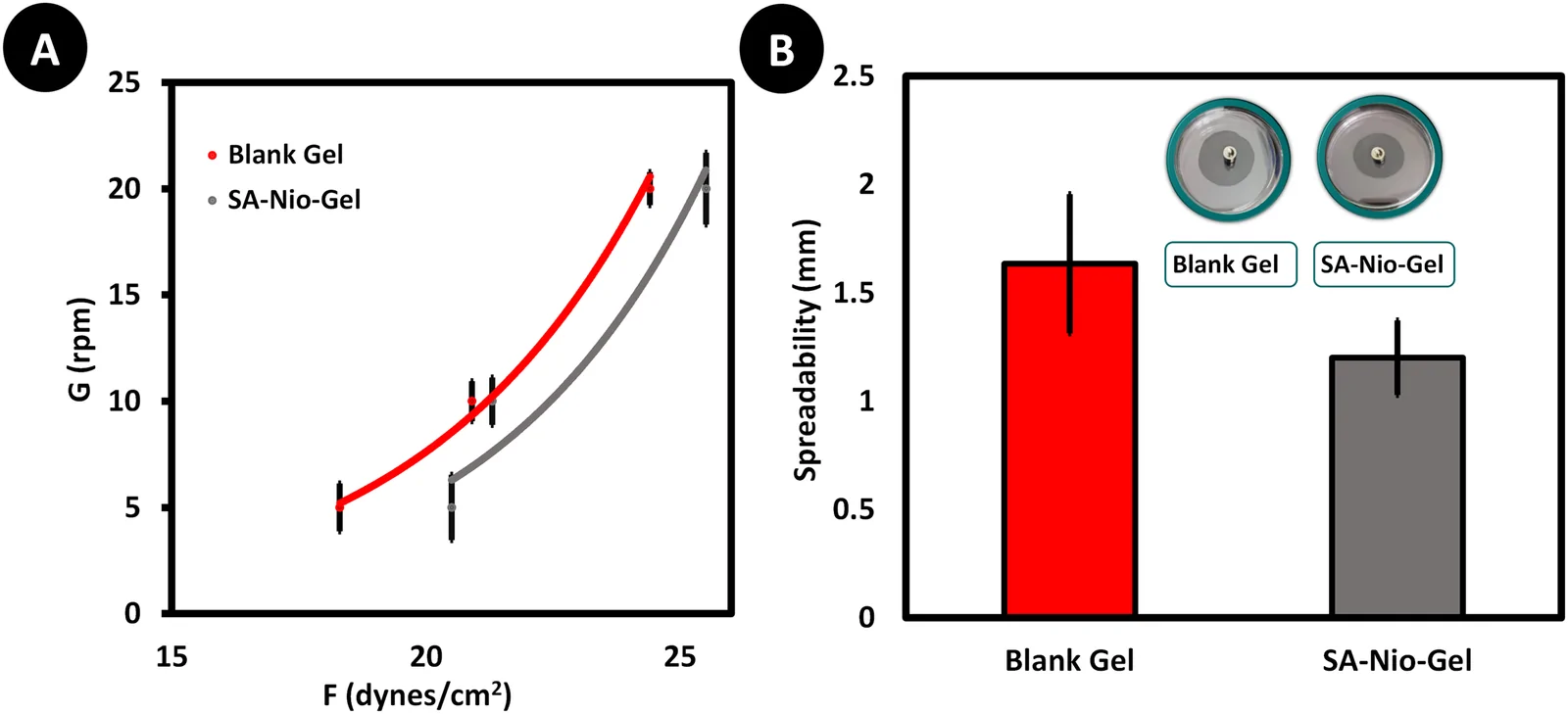
A) rheogram and B) spreadability analysis of blank Gel and SA-Nio-Gel.

### Spreadability test

The results of spreadability test are presented in Fig 3B. The SA-Nio-Gel formulation exhibited higher spreadability compared to the blank Gel; however, this difference was not significant (*P* > 0.05). This finding indicates that niosomes can be integrated into the gel network structure without disrupting it, allowing it to maintain its desired localized properties. If the formulation has poor spreadability, the drug will accumulate in one spot and other parts of the eye will not receive enough dose [40]. Inadequate spreadability over the ocular surface may also cause a foreign body sensation, burning, and irritation. In contrast, an easily spreadable gel can be applied with low shear stress, leading to better patient acceptance and improved treatment efficacy [39].

### In vitro Release

The release profiles of SA solution (Sol), SA-Nio and SA-Nio-Gel are illustrated in Fig 4A. According to the results, Sol showed the fastest release, reaching almost 100% release by the end of the study. The niosomal formulation reduced the drug release rate, reaching **approximately** 70% over 10 h. The niosomes act as a barrier and provide a more gradual release. SA-Nio-Gel exhibited a sustained-release profile, only 30% of the drug was released within the first 10 h. It was obvious that incorporation of SA-loaded niosomes into the gel effectively prevented the initial burst release and decreased the overall drug release rate. This effect is attributed to the dual-barrier system, comprising the niosomal bilayer and the polymeric gel network [41]. This is highly desirable for ocular therapy because it reduces dosing frequency through sustained drug release, thereby enhancing patient adherence and treatment efficacy [26]. Reducing the frequency of medication administration, particularly in ophthalmic treatments, is a key factor in improving patient adherence. The frequent instillation of eye drops, especially during long-term treatment regimens, can lead to missed or unintentionally omitted doses. Subsequently, the use of a drug delivery system capable of sustained release offers a promising approach to simplifying the therapeutic regimen and potentially enhancing patient adherence to treatment.

**Fig 4:**
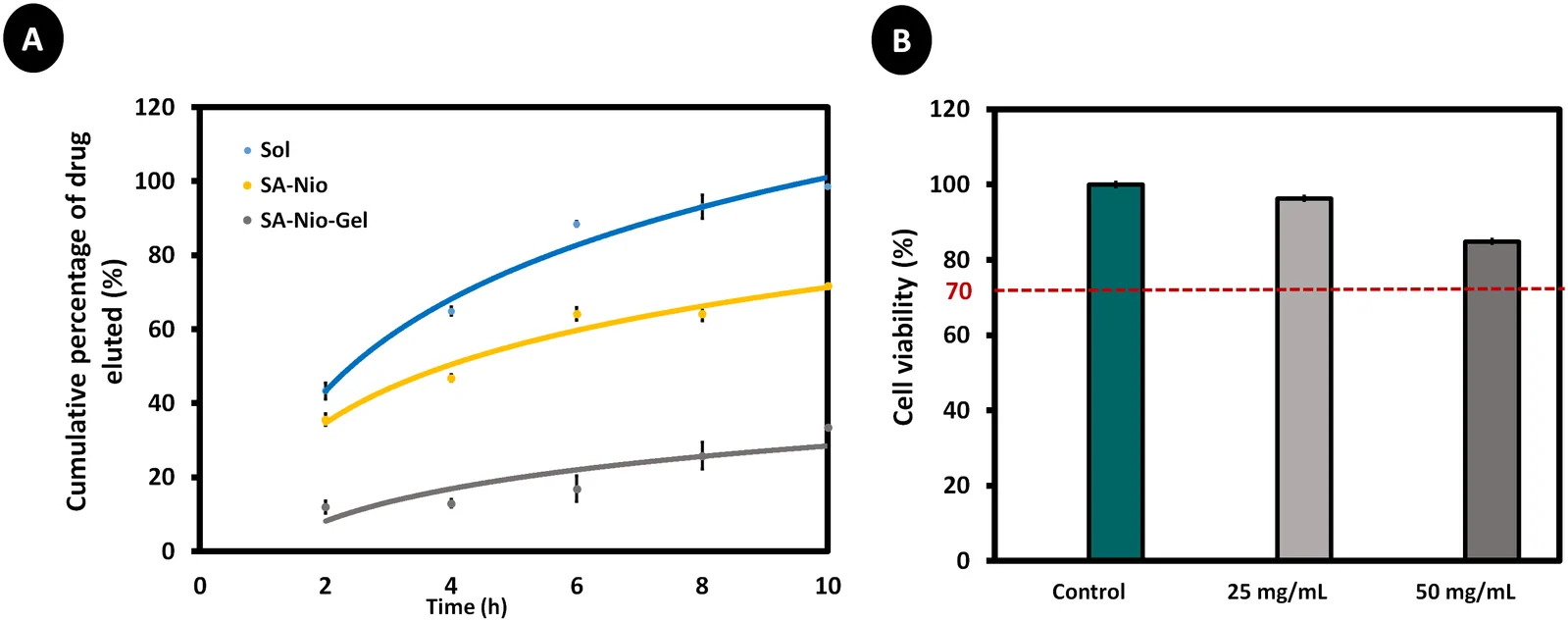
A) in-v*itro* release profile of Sol, SA-Nio and SA-Nio-Gel and B) cytotoxicity profile of gel extract in Hu02 cells (n = 3, mean ± SD).

### *In vitro* cell viability assay

In order to evaluate the cytotoxicity of optimal formulation, the MTT assay was performed. According to international standards such as ISO 10993−5, materials that exhibit more than 70% cell viability are considered non-toxic [42]. As shown in Fig 4B, cell viability remained significantly above the threshold throughout the experiment. Furthermore, viability following treatment with the formulation was comparable to the control group (untreated cells). Therefore, the formulation can be considered biocompatible and safe for ocular delivery. Ocular surface cells are highly sensitive to irritants and toxic substances, making biocompatibility essential in eye care. Moreover, reducing local discomfort improves patient adherence to treatment regimens. This finding is consistent with previous reports by Sangkana et al. and Lim et al., which showed that formulations based on similar biomaterials were non-toxic to cells [43,44].

### *In vitro* antibacterial activity

The antibacterial efficacy of SA-Nio and SA-Nio-Gel was evaluated against *S. aureus*. As illustrated in Fig 5A and 5B, SA-Nio-Gel displayed a smaller diameter of the growth inhibition zone compared to SA-Nio (*P* < 0.05). This lower antibacterial activity observed in the SA-Nio-Gel formulation is most likely due to an additional diffusional barrier. In the SA-Nio, the niosomes are in direct contact with the bacterial environment, allowing the released SA to reach its target *via* a short and rapid diffusion path. In the SA-Nio-Gel, the niosomes are incorporated within a polymeric network. This structure imposes a two-step release process: the antibiotic must first diffuse out of the niosomes and then pass through the viscous gel matrix before encountering the bacteria. This dual barrier significantly slows the overall release rate. These findings are consistent with the results of the drug release study, which clearly demonstrated a slower and more sustained release profile for SA-Nio-Gel compared to SA-Nio. Although this sustained release initially results in a lower antibacterial effect *in vitro*, it offers major advantages for ocular drug delivery. The gel matrix increases viscosity and mucoadhesion, extending the retention of the formulation on the ocular surface. At the same time, niosomes facilitate transcorneal penetration, improve drug permeation through the bacterial cell wall and protect the antibiotic from enzymatic degradation [41]. Thus, this sustained release may help maintain the antibiotic concentration on the ocular surface for an extended period, potentially enhancing the antibacterial activity against *S. aureus*. Together, these features ensure a controlled drug release that maintains therapeutic concentrations in the eye for an extended period, thereby reducing the frequency of administration and increasing treatment efficacy. Based on the findings of this study, SA-Nio-Gel may represent a promising approach for more sustained drug release while reducing the treatment burden associated with frequent administration. The proposed mechanism of the antibacterial activity of SA-Nio-Gel is schematically shown in Fig 5C.

**Fig 5:**
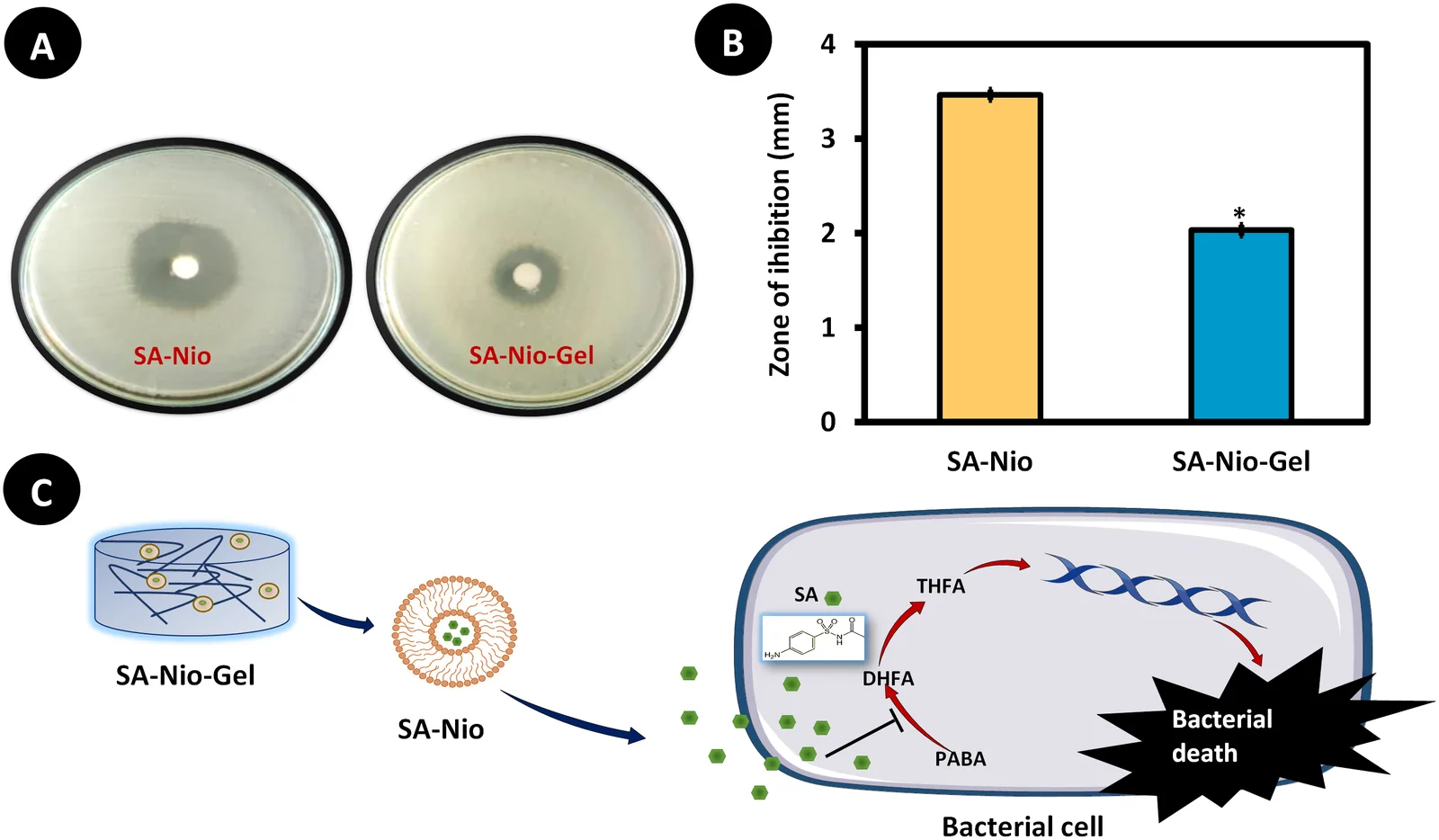
A) images of the inhibition zone, B) the diameter of inhibition zone around SA-Nio and SA-Nio-Gel (n = 3, mean ± SD) and C) schematic representation of antibacterial activity of SA-Nio-Gel in the bacterial cells.

### *Ex vivo* evaluation of ocular retention

As shown in Fig 6A, the Sol formulation was rapidly washed away from the ocular mucosa, resulting in negligible penetration into the mucosal tissue. In SA-Nio, niosomes provided a protective barrier and enhanced the penetration of SA into the ocular mucosa. These lipid nanocarriers can interact with the mucous layer of the eye, thereby prolonging the precorneal residence time of the loaded drug [45]. However, SA-Nio-Gel exhibited the minimal drainage and enhanced drug penetration compared to the Sol and SA-Nio primarily due to its mucoadhesive properties (*P* < 0.05). Prolonging the residence time of the drug on the ocular surface is crucial for effective ocular drug delivery, since rapid clearance of the drug through blinking and tear drainage significantly reduces its therapeutic efficacy [13]. According to the obtained results, combining niosomal carriers with a gel matrix could provide a promising strategy to overcome these physiological barriers and enhancing drug retention. A schematic illustration of the interaction of different formulations with the ocular mucosa is shown in Fig 6B.

**Fig 6:**
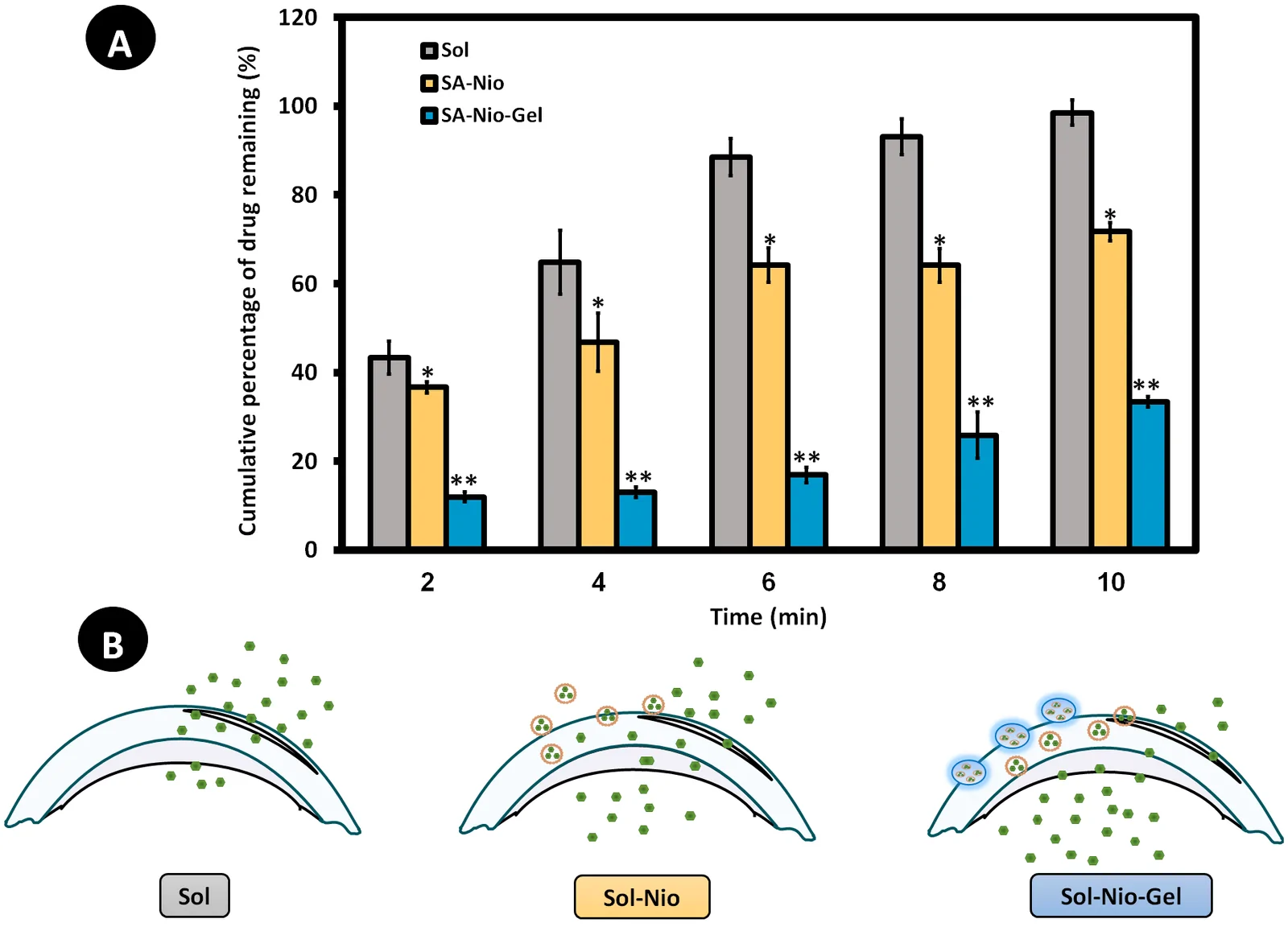
A) cumulative percentage of drug remaining on ocular mucosa and B) schematic illustration of the interaction of different formulations with the ocular mucosa.

## Conclusion

The results of this study indicate that SA-Nio-Gel may serve as a promising platform for ocular drug delivery. The formulation provides sustained release of SA, which may help maintain therapeutic drug levels in the eye. It also showed good biocompatibility, indicating its safety for ophthalmic application. Moreover, the formulation displayed potent antibacterial activity against *S. aureus*, confirming its therapeutic potential in the treatment of ocular infections. Compared with SA solution and SA-Nio, SA-Nio-Gel significantly reduced precorneal drainage and enhanced drug penetration across ocular mucosa. These findings highlight its potential as an effective approach for enhanced and sustained ocular delivery.
